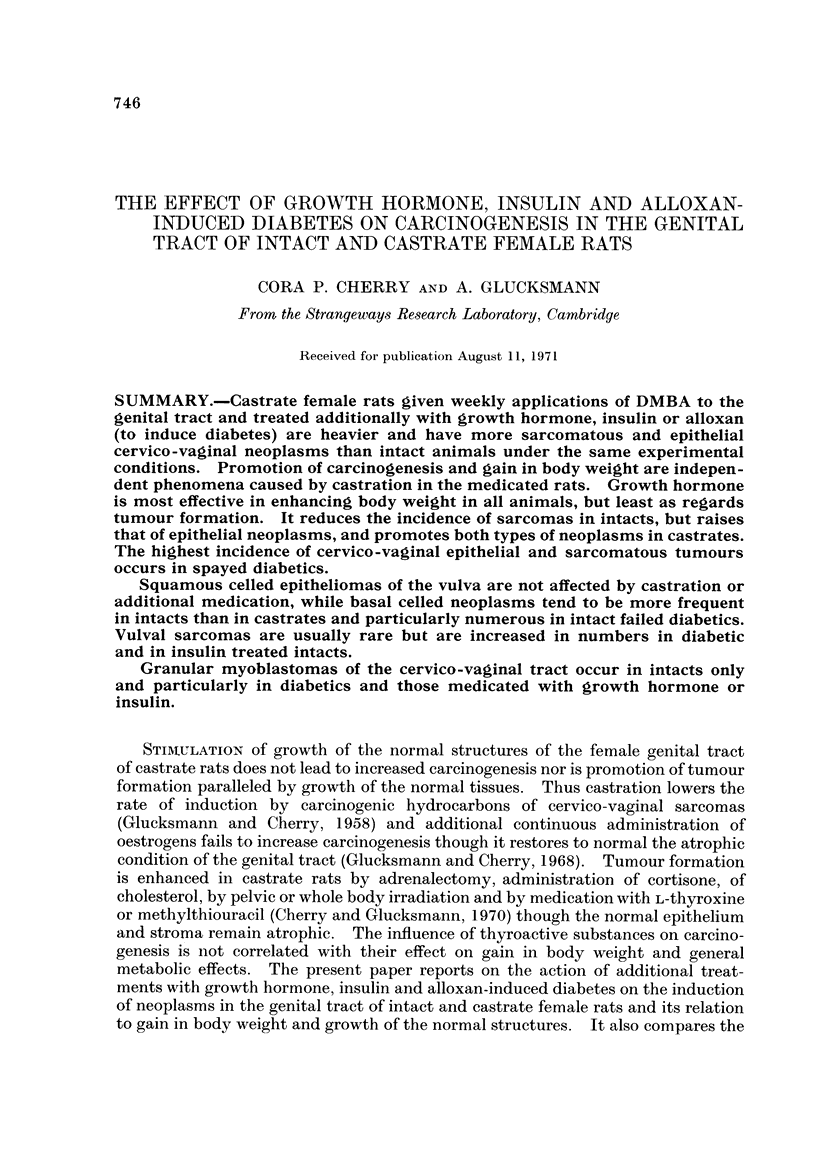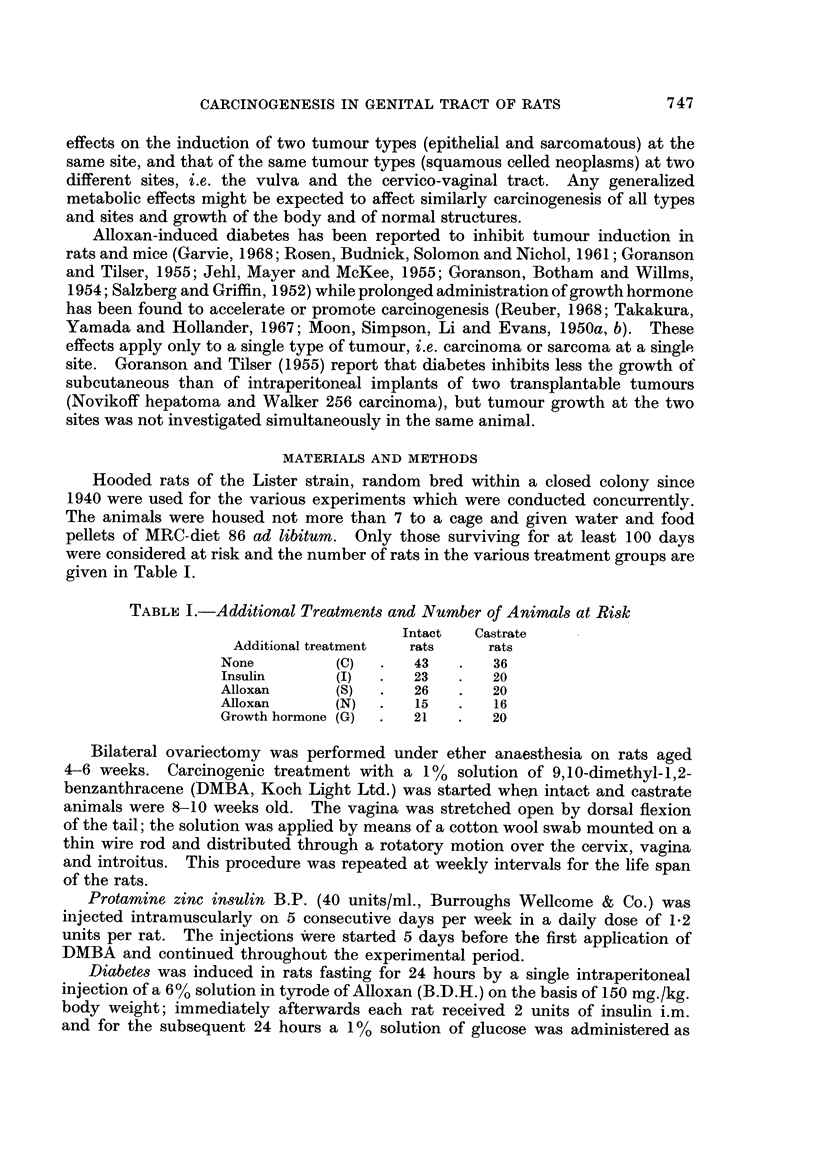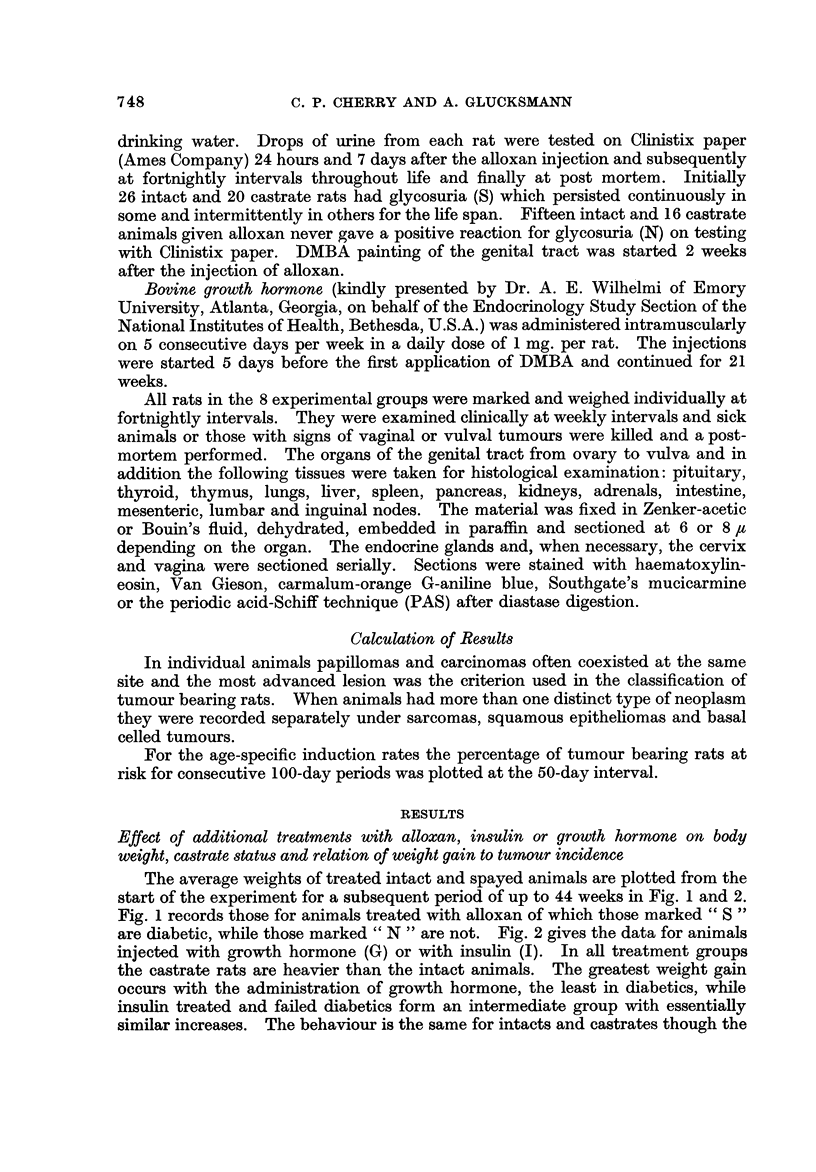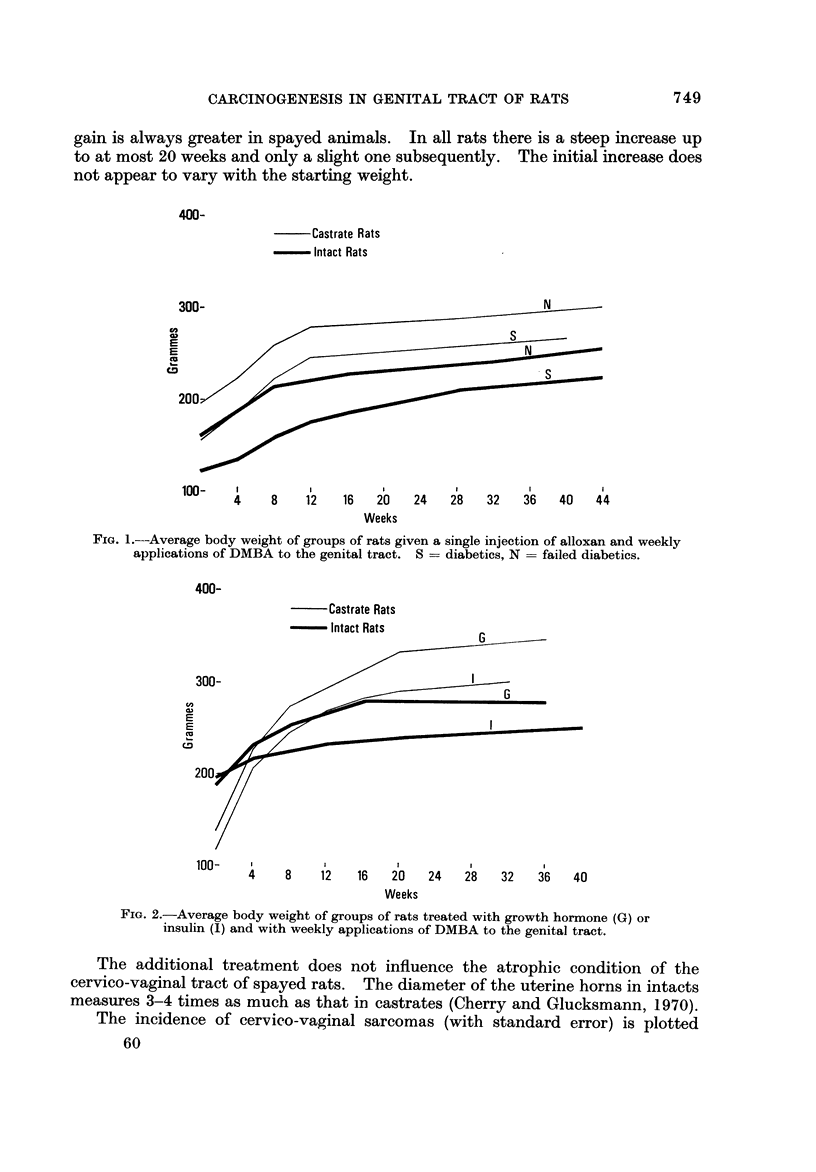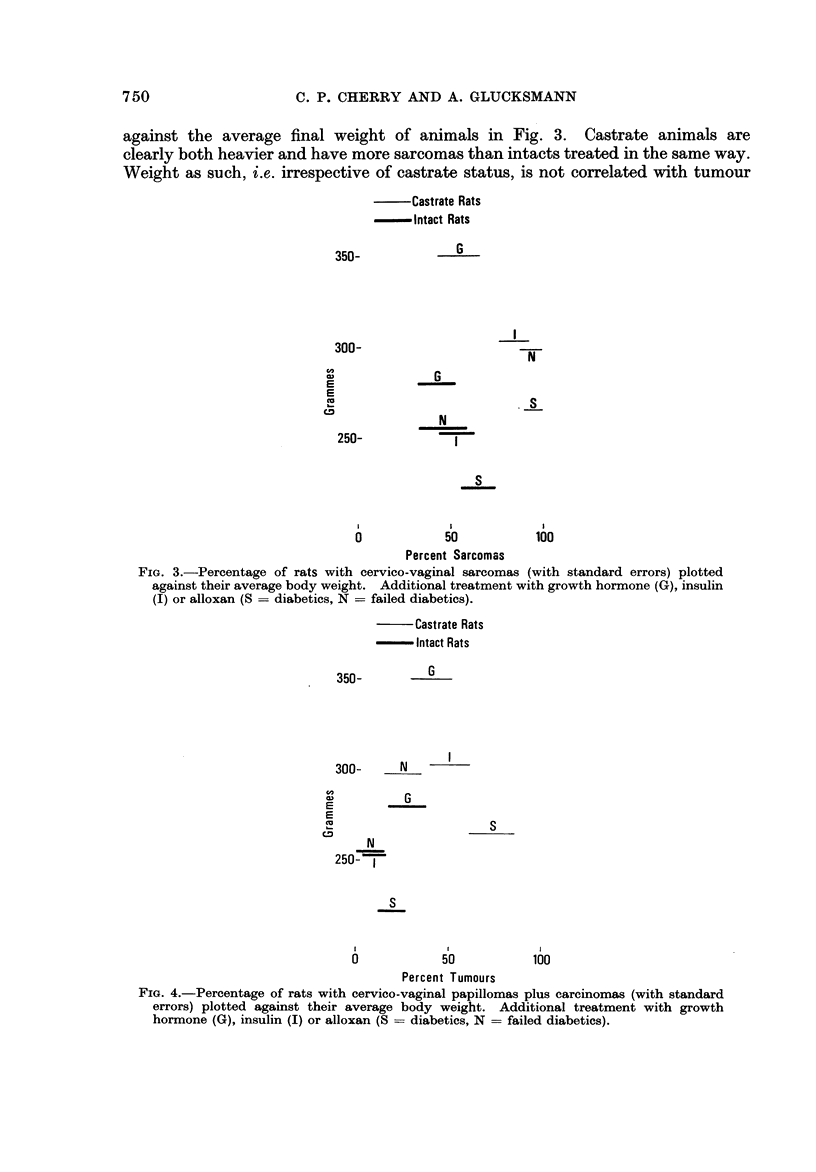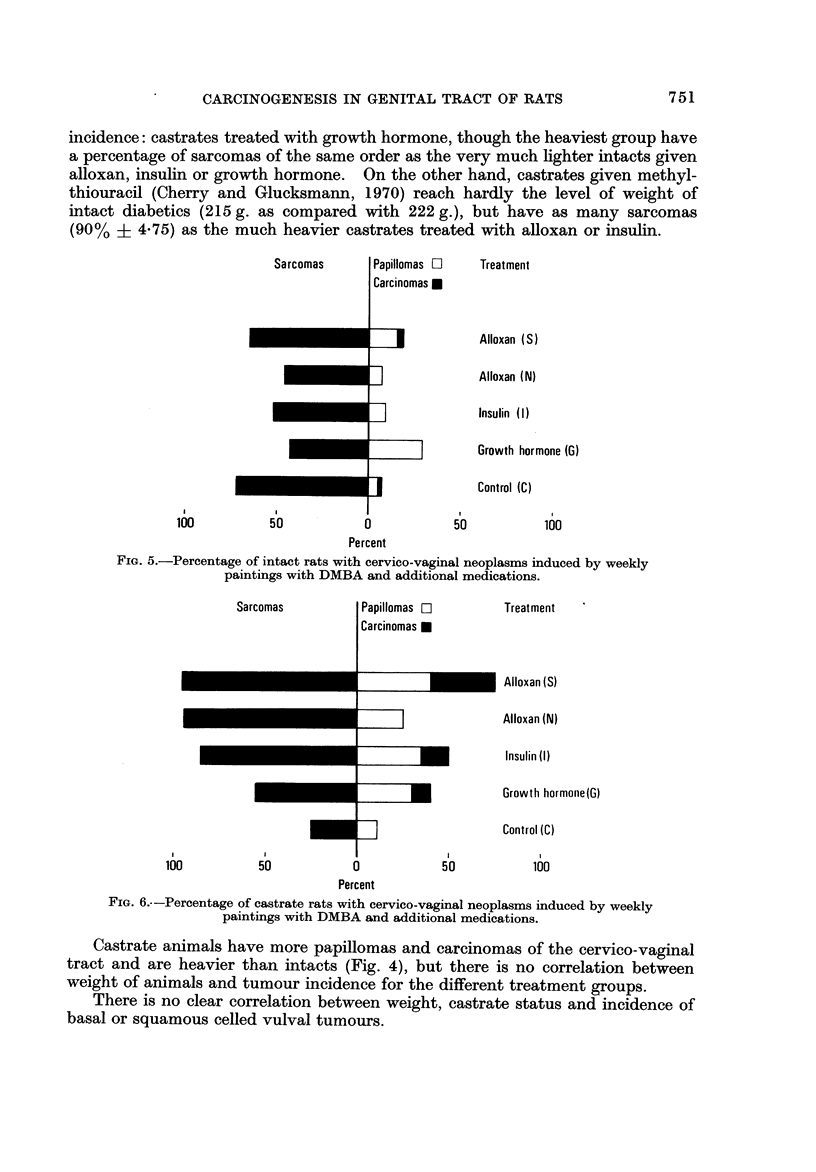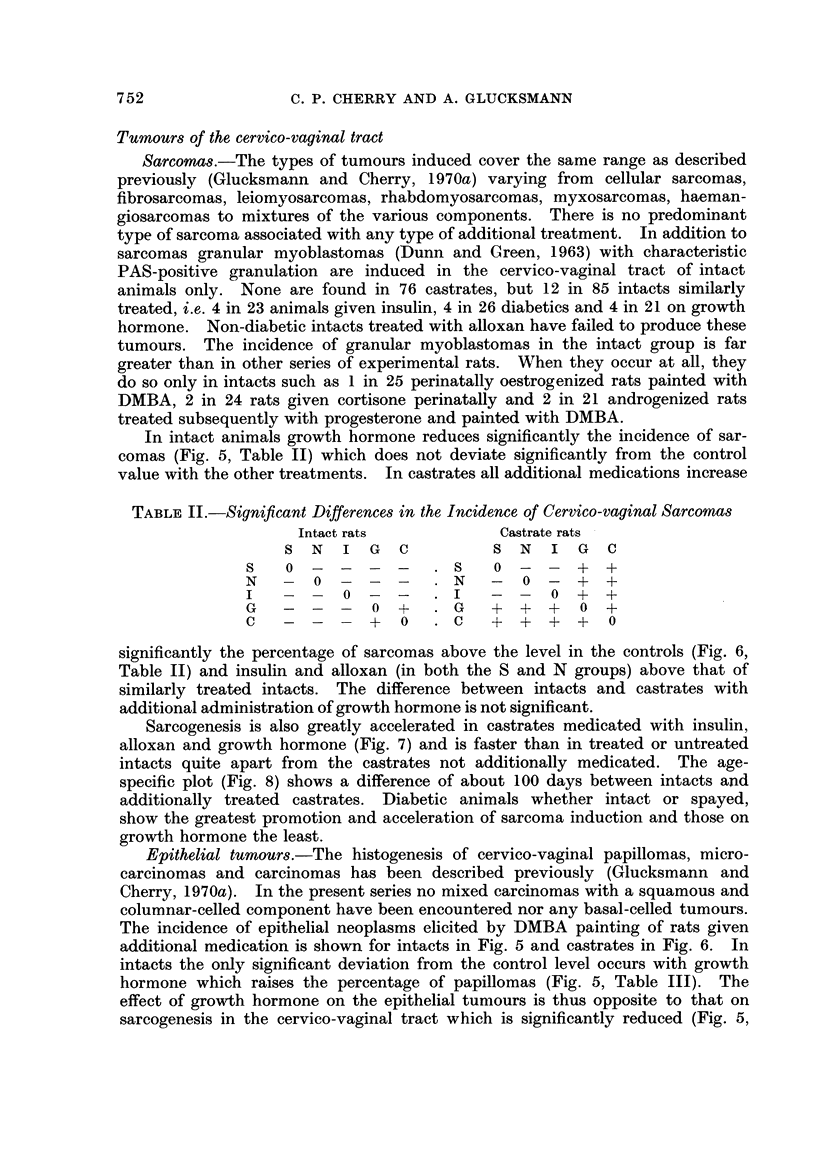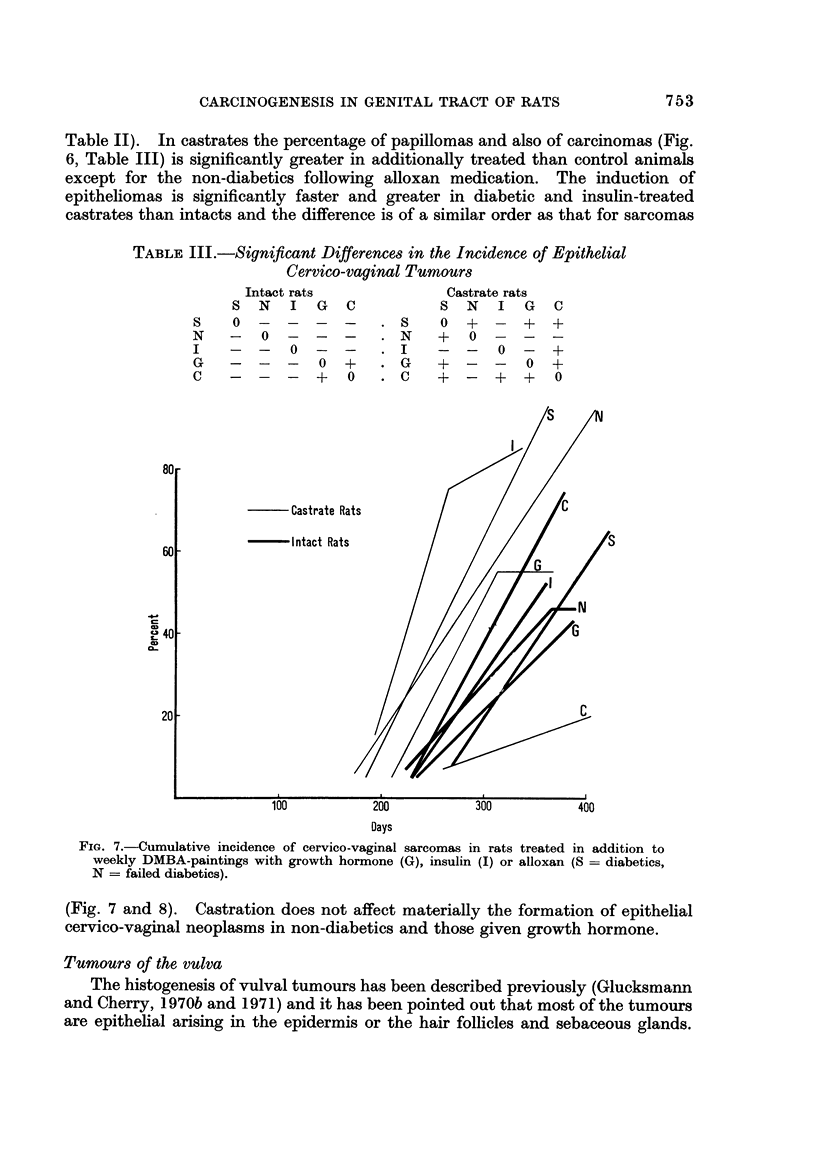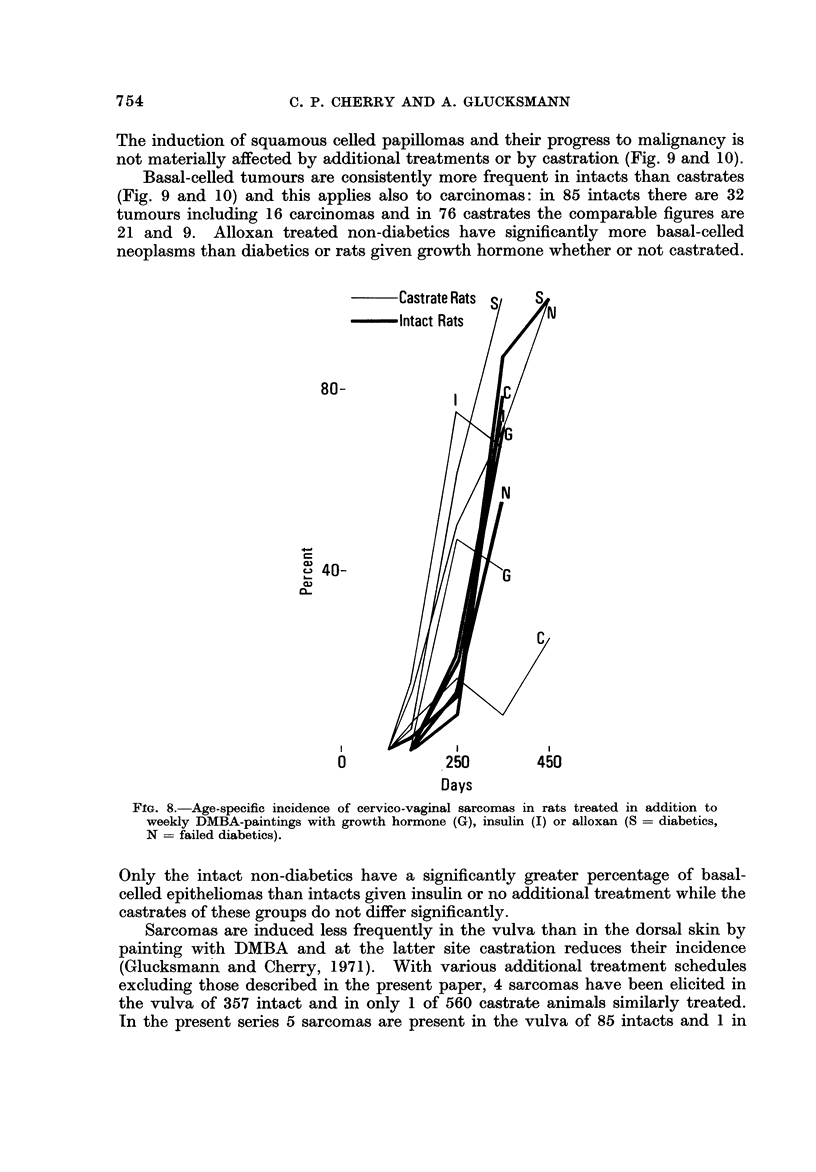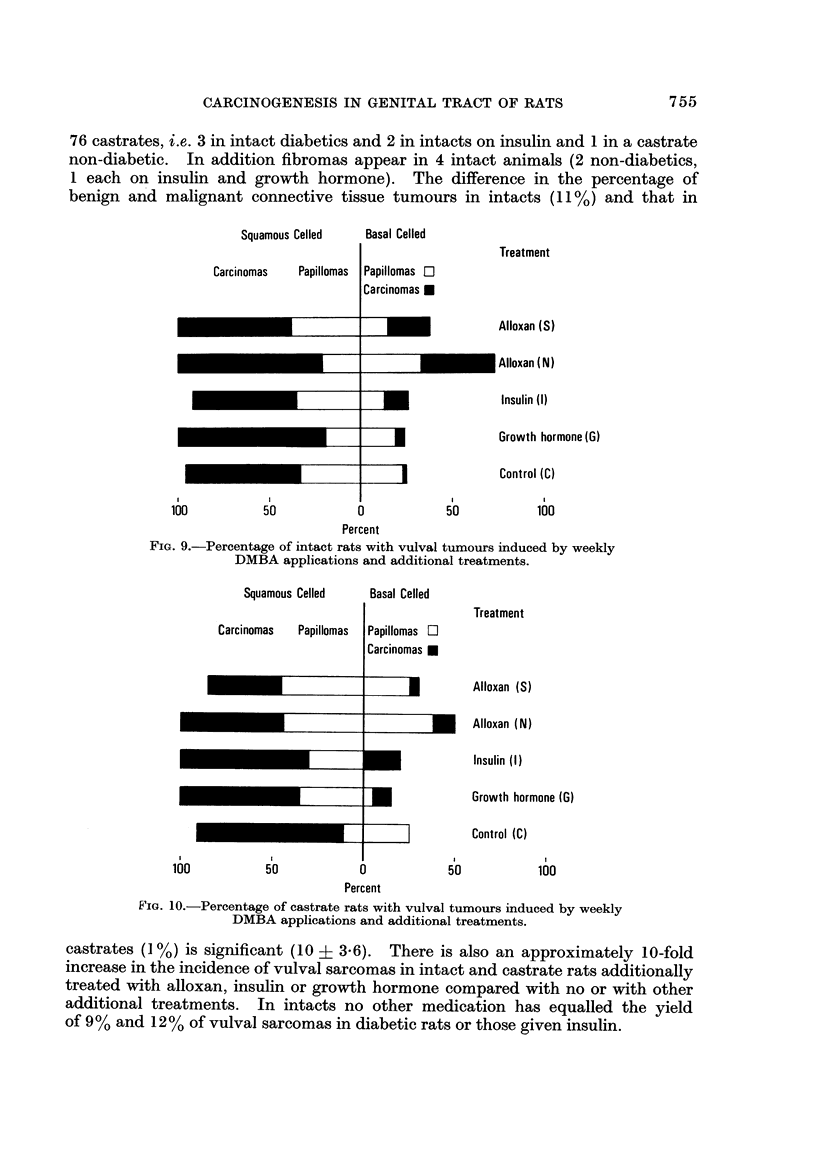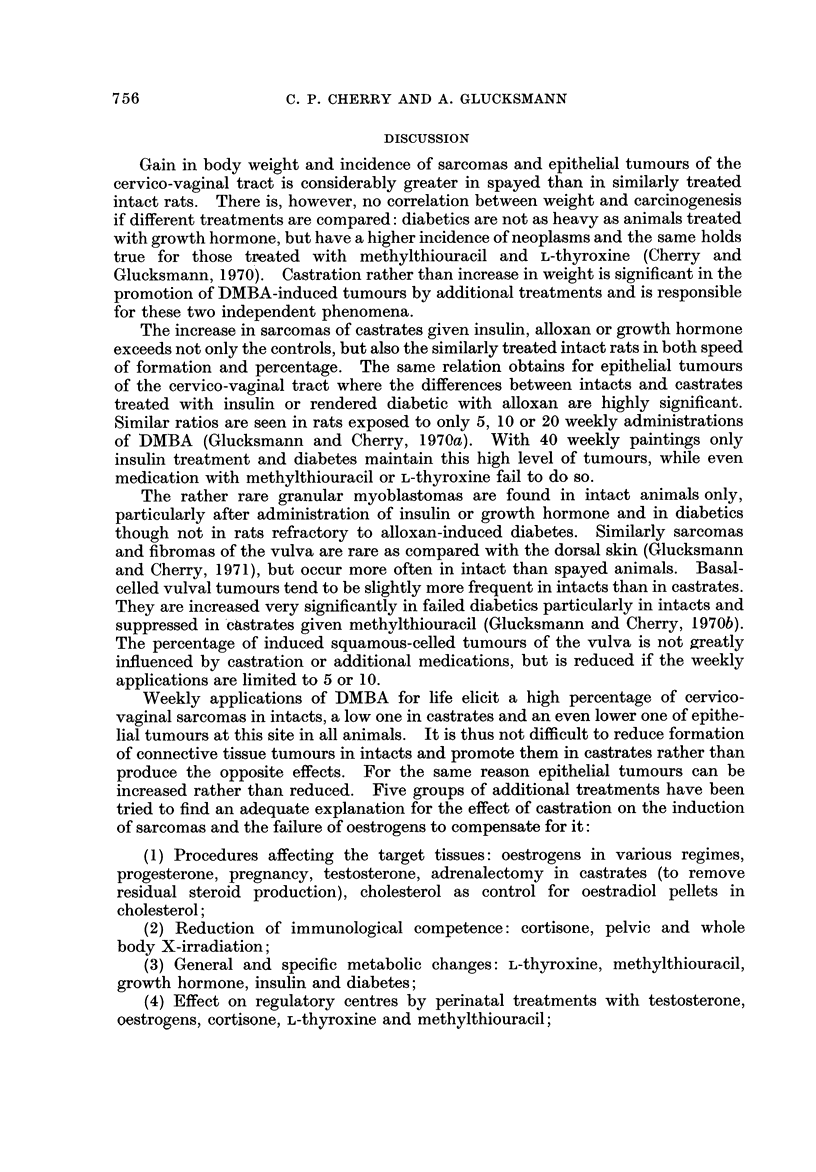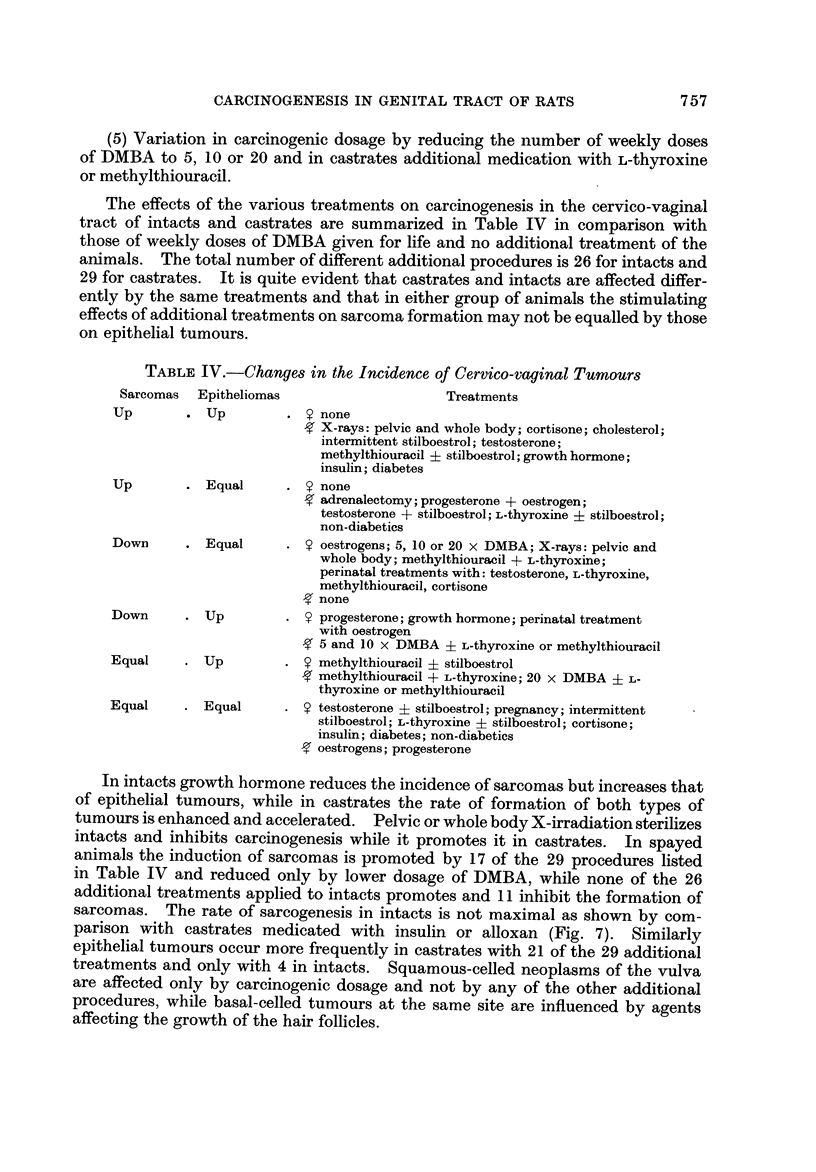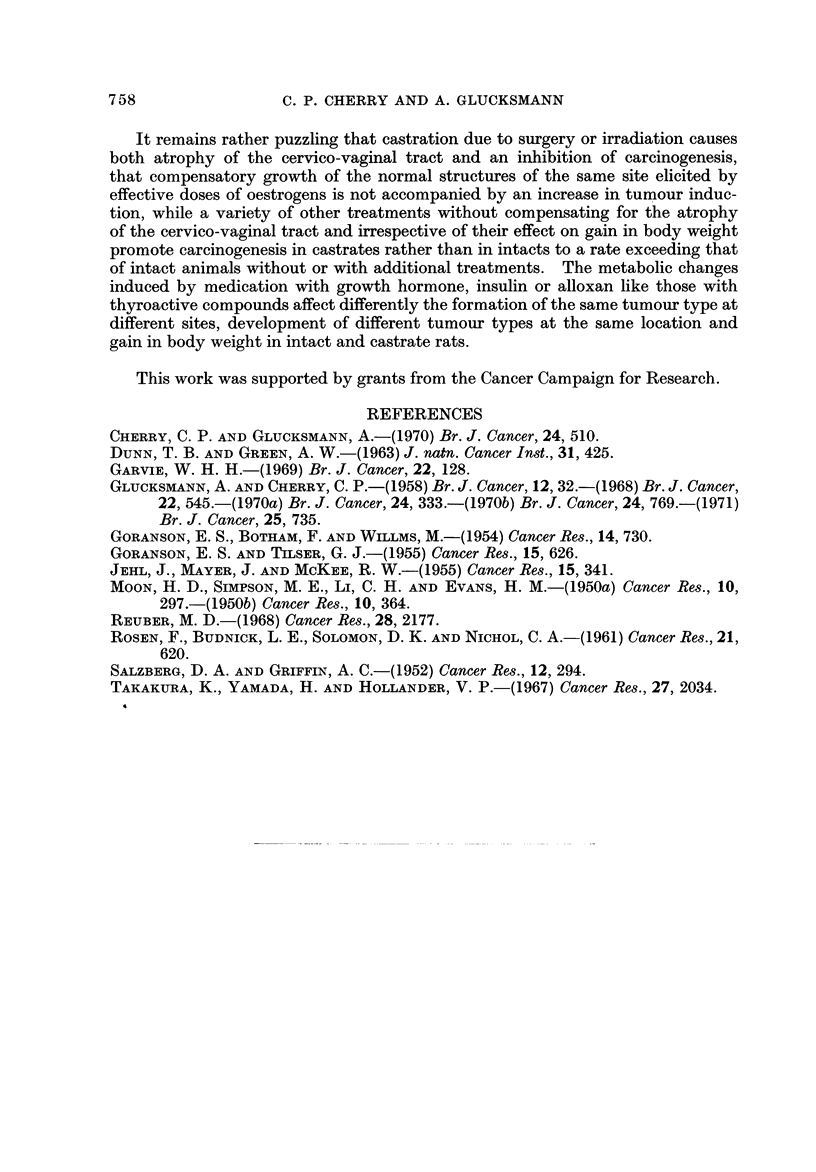# The Effect of Growth Hormone, Insulin and Alloxan-Induced Diabetes on Carcinogenesis in the Genital Tract of Intact and Castrate Female Rats

**DOI:** 10.1038/bjc.1971.89

**Published:** 1971-12

**Authors:** Cora P. Cherry, A. Glucksmann

## Abstract

Castrate female rats given weekly applications of DMBA to the genital tract and treated additionally with growth hormone, insulin or alloxan (to induce diabetes) are heavier and have more sarcomatous and epithelial cervico-vaginal neoplasms than intact animals under the same experimental conditions. Promotion of carcinogenesis and gain in body weight are independent phenomena caused by castration in the medicated rats. Growth hormone is most effective in enhancing body weight in all animals, but least as regards tumour formation. It reduces the incidence of sarcomas in intacts, but raises that of epithelial neoplasms, and promotes both types of neoplasms in castrates. The highest incidence of cervico-vaginal epithelial and sarcomatous tumours occurs in spayed diabetics.

Squamous celled epitheliomas of the vulva are not affected by castration or additional medication, while basal celled neoplasms tend to be more frequent in intacts than in castrates and particularly numerous in intact failed diabetics. Vulval sarcomas are usually rare but are increased in numbers in diabetic and in insulin treated intacts.

Granular myoblastomas of the cervico-vaginal tract occur in intacts only and particularly in diabetics and those medicated with growth hormone or insulin.


### THE EFFECT OF GROWTH HORMONE, INSULIN AND ALLOXAN-INDUCED DIABETES ON CARCINOGENESIS IN THE GENITAL TRACT OF INTACT AND CASTRATE FEMALE RATS

CORA P. CHERRY AND A. GLUCKSMANN

*From the Strangeways Research Laboratory, Cambridge*

Received for publication August 11, 1971

**SUMMARY.—Castrate female rats given weekly applications of DMBA to the genital tract and treated additionally with growth hormone, insulin or alloxan (to induce diabetes) are heavier and have more sarcomatous and epithelial cervico-vaginal neoplasms than intact animals under the same experimental conditions. Promotion of carcinogenesis and gain in body weight are independent phenomena caused by castration in the medicated rats. Growth hormone is most effective in enhancing body weight in all animals, but least as regards tumour formation. It reduces the incidence of sarcomas in intacts, but raises that of epithelial neoplasms, and promotes both types of neoplasms in castrates. The highest incidence of cervico-vaginal epithelial and sarcomatous tumours occurs in spayed diabetics.**

**Squamous celled epitheliomas of the vulva are not affected by castration or additional medication, while basal celled neoplasms tend to be more frequent in intacts than in castrates and particularly numerous in intact failed diabetics. Vulval sarcomas are usually rare but are increased in numbers in diabetic and in insulin treated intacts.**

**Granular myoblastomas of the cervico-vaginal tract occur in intacts only and particularly in diabetics and those medicated with growth hormone or insulin.**

STIMULATION of growth of the normal structures of the female genital tract of castrate rats does not lead to increased carcinogenesis nor is promotion of tumour formation paralleled by growth of the normal tissues. Thus castration lowers the rate of induction by carcinogenic hydrocarbons of cervico-vaginal sarcomas (Glucksmann and Cherry, 1958) and additional continuous administration of oestrogens fails to increase carcinogenesis though it restores to normal the atrophic condition of the genital tract (Glucksmann and Cherry, 1968). Tumour formation is enhanced in castrate rats by adrenalectomy, administration of cortisone, of cholesterol, by pelvic or whole body irradiation and by medication with L-thyroxine or methylthiouracil (Cherry and Glucksmann, 1970) though the normal epithelium and stroma remain atrophic. The influence of thyroactive substances on carcinogenesis is not correlated with their effect on gain in body weight and general metabolic effects. The present paper reports on the action of additional treatments with growth hormone, insulin and alloxan-induced diabetes on the induction of neoplasms in the genital tract of intact and castrate female rats and its relation to gain in body weight and growth of the normal structures. It also compares the effects on the induction of two tumour types (epithelial and sarcomatous) at the same site, and that of the same tumour types (squamous celled neoplasms) at two different sites, *i.e.* the vulva and the cervico-vaginal tract. Any generalized metabolic effects might be expected to affect similarly carcinogenesis of all types and sites and growth of the body and of normal structures.

Alloxan-induced diabetes has been reported to inhibit tumour induction in rats and mice (Garvie, 1968; Rosen, Budnick, Solomon and Nichol, 1961; Goranson and Tilser, 1955; Jehl, Mayer and McKee, 1955; Goranson, Botham and Willms, 1954; Salzberg and Griffin, 1952) while prolonged administration of growth hormone has been found to accelerate or promote carcinogenesis (Reuber, 1968; Takakura, Yamada and Hollander, 1967; Moon, Simpson, Li and Evans, 1950*a*, *b*). These effects apply only to a single type of tumour, *i.e.* carcinoma or sarcoma at a single site. Goranson and Tilser (1955) report that diabetes inhibits less the growth of subcutaneous than of intraperitoneal implants of two transplantable tumours (Novikoff hepatoma and Walker 256 carcinoma), but tumour growth at the two sites was not investigated simultaneously in the same animal.

### MATERIALS AND METHODS

Hooded rats of the Lister strain, random bred within a closed colony since 1940 were used for the various experiments which were conducted concurrently. The animals were housed not more than 7 to a cage and given water and food pellets of MRC-diet 86 *ad libitum.* Only those surviving for at least 100 days were considered at risk and the number of rats in the various treatment groups are given in Table I.

TABLE I.—*Additional Treatments and Number of Animals at Risk*

| Additional treatment | | Intact rats | Castrate rats |
|---|---|---|---|
| None | (C) | 43 | 36 |
| Insulin | (I) | 23 | 20 |
| Alloxan | (S) | 26 | 20 |
| Alloxan | (N) | 15 | 16 |
| Growth hormone | (G) | 21 | 20 |

Bilateral ovariectomy was performed under ether anaesthesia on rats aged 4–6 weeks. Carcinogenic treatment with a 1% solution of 9,10-dimethyl-1,2-benzanthracene (DMBA, Koch Light Ltd.) was started when intact and castrate animals were 8–10 weeks old. The vagina was stretched open by dorsal flexion of the tail; the solution was applied by means of a cotton wool swab mounted on a thin wire rod and distributed through a rotatory motion over the cervix, vagina and introitus. This procedure was repeated at weekly intervals for the life span of the rats.

*Protamine zinc insulin* B.P. (40 units/ml., Burroughs Wellcome & Co.) was injected intramuscularly on 5 consecutive days per week in a daily dose of 1·2 units per rat. The injections were started 5 days before the first application of DMBA and continued throughout the experimental period.

*Diabetes* was induced in rats fasting for 24 hours by a single intraperitoneal injection of a 6% solution in tyrode of Alloxan (B.D.H.) on the basis of 150 mg./kg. body weight; immediately afterwards each rat received 2 units of insulin i.m. and for the subsequent 24 hours a 1% solution of glucose was administered as drinking water. Drops of urine from each rat were tested on Clinistix paper (Ames Company) 24 hours and 7 days after the alloxan injection and subsequently at fortnightly intervals throughout life and finally at post mortem. Initially 26 intact and 20 castrate rats had glycosuria (S) which persisted continuously in some and intermittently in others for the life span. Fifteen intact and 16 castrate animals given alloxan never gave a positive reaction for glycosuria (N) on testing with Clinistix paper. DMBA painting of the genital tract was started 2 weeks after the injection of alloxan.

*Bovine growth hormone* (kindly presented by Dr. A. E. Wilhelmi of Emory University, Atlanta, Georgia, on behalf of the Endocrinology Study Section of the National Institutes of Health, Bethesda, U.S.A.) was administered intramuscularly on 5 consecutive days per week in a daily dose of 1 mg. per rat. The injections were started 5 days before the first application of DMBA and continued for 21 weeks.

All rats in the 8 experimental groups were marked and weighed individually at fortnightly intervals. They were examined clinically at weekly intervals and sick animals or those with signs of vaginal or vulval tumours were killed and a post-mortem performed. The organs of the genital tract from ovary to vulva and in addition the following tissues were taken for histological examination: pituitary, thyroid, thymus, lungs, liver, spleen, pancreas, kidneys, adrenals, intestine, mesenteric, lumbar and inguinal nodes. The material was fixed in Zenker-acetic or Bouin's fluid, dehydrated, embedded in paraffin and sectioned at 6 or 8 $\mu$ depending on the organ. The endocrine glands and, when necessary, the cervix and vagina were sectioned serially. Sections were stained with haematoxylin-eosin, Van Gieson, carmalum-orange G-aniline blue, Southgate's mucicarmine or the periodic acid-Schiff technique (PAS) after diastase digestion.

### *Calculation of Results*

In individual animals papillomas and carcinomas often coexisted at the same site and the most advanced lesion was the criterion used in the classification of tumour bearing rats. When animals had more than one distinct type of neoplasm they were recorded separately under sarcomas, squamous epitheliomas and basal celled tumours.

For the age-specific induction rates the percentage of tumour bearing rats at risk for consecutive 100-day periods was plotted at the 50-day interval.

### RESULTS

### *Effect of additional treatments with alloxan, insulin or growth hormone on body weight, castrate status and relation of weight gain to tumour incidence*

The average weights of treated intact and spayed animals are plotted from the start of the experiment for a subsequent period of up to 44 weeks in Fig. 1 and 2. Fig. 1 records those for animals treated with alloxan of which those marked " S " are diabetic, while those marked " N " are not. Fig. 2 gives the data for animals injected with growth hormone (G) or with insulin (I). In all treatment groups the castrate rats are heavier than the intact animals. The greatest weight gain occurs with the administration of growth hormone, the least in diabetics, while insulin treated and failed diabetics form an intermediate group with essentially similar increases. The behaviour is the same for intacts and castrates though the gain is always greater in spayed animals. In all rats there is a steep increase up to at most 20 weeks and only a slight one subsequently. The initial increase does not appear to vary with the starting weight.

FIG. 1.—Average body weight of groups of rats given a single injection of alloxan and weekly applications of DMBA to the genital tract. S = diabetics, N = failed diabetics.

FIG. 2.—Average body weight of groups of rats treated with growth hormone (G) or insulin (I) and with weekly applications of DMBA to the genital tract.

The additional treatment does not influence the atrophic condition of the cervico-vaginal tract of spayed rats. The diameter of the uterine horns in intacts measures 3–4 times as much as that in castrates (Cherry and Glucksmann, 1970).

The incidence of cervico-vaginal sarcomas (with standard error) is plotted against the average final weight of animals in Fig. 3. Castrate animals are clearly both heavier and have more sarcomas than intacts treated in the same way. Weight as such, *i.e.* irrespective of castrate status, is not correlated with tumour

FIG. 3.—Percentage of rats with cervico-vaginal sarcomas (with standard errors) plotted against their average body weight. Additional treatment with growth hormone (G), insulin (I) or alloxan (S = diabetics, N = failed diabetics).

FIG. 4.—Percentage of rats with cervico-vaginal papillomas plus carcinomas (with standard errors) plotted against their average body weight. Additional treatment with growth hormone (G), insulin (I) or alloxan (S = diabetics, N = failed diabetics).

incidence: castrates treated with growth hormone, though the heaviest group have a percentage of sarcomas of the same order as the very much lighter intacts given alloxan, insulin or growth hormone. On the other hand, castrates given methylthiouracil (Cherry and Glucksmann, 1970) reach hardly the level of weight of intact diabetics (215 g. as compared with 222 g.), but have as many sarcomas (90% $\pm$ 4·75) as the much heavier castrates treated with alloxan or insulin.

FIG. 5.—Percentage of intact rats with cervico-vaginal neoplasms induced by weekly paintings with DMBA and additional medications.

FIG. 6.—Percentage of castrate rats with cervico-vaginal neoplasms induced by weekly paintings with DMBA and additional medications.

Castrate animals have more papillomas and carcinomas of the cervico-vaginal tract and are heavier than intacts (Fig. 4), but there is no correlation between weight of animals and tumour incidence for the different treatment groups.

There is no clear correlation between weight, castrate status and incidence of basal or squamous celled vulval tumours.

*Tumours of the cervico-vaginal tract*

*Sarcomas.*—The types of tumours induced cover the same range as described previously (Glucksmann and Cherry, 1970*a*) varying from cellular sarcomas, fibrosarcomas, leiomyosarcomas, rhabdomyosarcomas, myxosarcomas, haemangiosarcomas to mixtures of the various components. There is no predominant type of sarcoma associated with any type of additional treatment. In addition to sarcomas granular myoblastomas (Dunn and Green, 1963) with characteristic PAS-positive granulation are induced in the cervico-vaginal tract of intact animals only. None are found in 76 castrates, but 12 in 85 intacts similarly treated, *i.e.* 4 in 23 animals given insulin, 4 in 26 diabetics and 4 in 21 on growth hormone. Non-diabetic intacts treated with alloxan have failed to produce these tumours. The incidence of granular myoblastomas in the intact group is far greater than in other series of experimental rats. When they occur at all, they do so only in intacts such as 1 in 25 perinatally oestrogenized rats painted with DMBA, 2 in 24 rats given cortisone perinatally and 2 in 21 androgenized rats treated subsequently with progesterone and painted with DMBA.

In intact animals growth hormone reduces significantly the incidence of sarcomas (Fig. 5, Table II) which does not deviate significantly from the control value with the other treatments. In castrates all additional medications increase

TABLE II.—*Significant Differences in the Incidence of Cervico-vaginal Sarcomas*

| Intact rats | S | N | I | G | C |
|---|---|---|---|---|---|
| S | 0 | — | — | — | — |
| N | — | 0 | — | — | — |
| I | — | — | 0 | — | — |
| G | — | — | — | 0 | + |
| C | — | — | — | + | 0 |

| Castrate rats | S | N | I | G | C |
|---|---|---|---|---|---|
| S | 0 | — | — | + | + |
| N | — | 0 | — | + | + |
| I | — | — | 0 | + | + |
| G | + | + | + | 0 | + |
| C | + | + | + | + | 0 |

significantly the percentage of sarcomas above the level in the controls (Fig. 6, Table II) and insulin and alloxan (in both the S and N groups) above that of similarly treated intacts. The difference between intacts and castrates with additional administration of growth hormone is not significant.

Sarcogenesis is also greatly accelerated in castrates medicated with insulin, alloxan and growth hormone (Fig. 7) and is faster than in treated or untreated intacts quite apart from the castrates not additionally medicated. The age-specific plot (Fig. 8) shows a difference of about 100 days between intacts and additionally treated castrates. Diabetic animals whether intact or spayed, show the greatest promotion and acceleration of sarcoma induction and those on growth hormone the least.

*Epithelial tumours.*—The histogenesis of cervico-vaginal papillomas, microcarcinomas and carcinomas has been described previously (Glucksmann and Cherry, 1970*a*). In the present series no mixed carcinomas with a squamous and columnar-celled component have been encountered nor any basal-celled tumours. The incidence of epithelial neoplasms elicited by DMBA painting of rats given additional medication is shown for intacts in Fig. 5 and castrates in Fig. 6. In intacts the only significant deviation from the control level occurs with growth hormone which raises the percentage of papillomas (Fig. 5, Table III). The effect of growth hormone on the epithelial tumours is thus opposite to that on sarcogenesis in the cervico-vaginal tract which is significantly reduced (Fig. 5,

Table II). In castrates the percentage of papillomas and also of carcinomas (Fig. 6, Table III) is significantly greater in additionally treated than control animals except for the non-diabetics following alloxan medication. The induction of epitheliomas is significantly faster and greater in diabetic and insulin-treated castrates than intacts and the difference is of a similar order as that for sarcomas

TABLE III.—*Significant Differences in the Incidence of Epithelial Cervico-vaginal Tumours*

| | Intact rats | | | | | | | Castrate rats | | | | |
|---|---|---|---|---|---|---|---|---|---|---|---|---|
| | S | N | I | G | C | | | S | N | I | G | C |
| S | 0 | – | – | – | – | . | S | 0 | + | – | + | + |
| N | – | 0 | – | – | – | . | N | + | 0 | – | – | – |
| I | – | – | 0 | – | – | . | I | – | – | 0 | – | + |
| G | – | – | – | 0 | + | . | G | + | – | – | 0 | + |
| C | – | – | – | + | 0 | . | C | + | – | + | + | 0 |

FIG. 7.—Cumulative incidence of cervico-vaginal sarcomas in rats treated in addition to weekly DMBA-paintings with growth hormone (G), insulin (I) or alloxan (S = diabetics, N = failed diabetics).

(Fig. 7 and 8). Castration does not affect materially the formation of epithelial cervico-vaginal neoplasms in non-diabetics and those given growth hormone.

*Tumours of the vulva*

The histogenesis of vulval tumours has been described previously (Glucksmann and Cherry, 1970*b* and 1971) and it has been pointed out that most of the tumours are epithelial arising in the epidermis or the hair follicles and sebaceous glands.

The induction of squamous celled papillomas and their progress to malignancy is not materially affected by additional treatments or by castration (Fig. 9 and 10).

Basal-celled tumours are consistently more frequent in intacts than castrates (Fig. 9 and 10) and this applies also to carcinomas: in 85 intacts there are 32 tumours including 16 carcinomas and in 76 castrates the comparable figures are 21 and 9. Alloxan treated non-diabetics have significantly more basal-celled neoplasms than diabetics or rats given growth hormone whether or not castrated.

FIG. 8.—Age-specific incidence of cervico-vaginal sarcomas in rats treated in addition to weekly DMBA-paintings with growth hormone (G), insulin (I) or alloxan (S = diabetics, N = failed diabetics).

Only the intact non-diabetics have a significantly greater percentage of basal-celled epitheliomas than intacts given insulin or no additional treatment while the castrates of these groups do not differ significantly.

Sarcomas are induced less frequently in the vulva than in the dorsal skin by painting with DMBA and at the latter site castration reduces their incidence (Glucksmann and Cherry, 1971). With various additional treatment schedules excluding those described in the present paper, 4 sarcomas have been elicited in the vulva of 357 intact and in only 1 of 560 castrate animals similarly treated. In the present series 5 sarcomas are present in the vulva of 85 intacts and 1 in

76 castrates, *i.e.* 3 in intact diabetics and 2 in intacts on insulin and 1 in a castrate non-diabetic. In addition fibromas appear in 4 intact animals (2 non-diabetics, 1 each on insulin and growth hormone). The difference in the percentage of benign and malignant connective tissue tumours in intacts (11%) and that in

FIG. 9.—Percentage of intact rats with vulval tumours induced by weekly DMBA applications and additional treatments.

FIG. 10.—Percentage of castrate rats with vulval tumours induced by weekly DMBA applications and additional treatments.

castrates (1%) is significant (10 $\pm$ 3·6). There is also an approximately 10-fold increase in the incidence of vulval sarcomas in intact and castrate rats additionally treated with alloxan, insulin or growth hormone compared with no or with other additional treatments. In intacts no other medication has equalled the yield of 9% and 12% of vulval sarcomas in diabetic rats or those given insulin.

### DISCUSSION

Gain in body weight and incidence of sarcomas and epithelial tumours of the cervico-vaginal tract is considerably greater in spayed than in similarly treated intact rats. There is, however, no correlation between weight and carcinogenesis if different treatments are compared: diabetics are not as heavy as animals treated with growth hormone, but have a higher incidence of neoplasms and the same holds true for those treated with methylthiouracil and L-thyroxine (Cherry and Glucksmann, 1970). Castration rather than increase in weight is significant in the promotion of DMBA-induced tumours by additional treatments and is responsible for these two independent phenomena.

The increase in sarcomas of castrates given insulin, alloxan or growth hormone exceeds not only the controls, but also the similarly treated intact rats in both speed of formation and percentage. The same relation obtains for epithelial tumours of the cervico-vaginal tract where the differences between intacts and castrates treated with insulin or rendered diabetic with alloxan are highly significant. Similar ratios are seen in rats exposed to only 5, 10 or 20 weekly administrations of DMBA (Glucksmann and Cherry, 1970*a*). With 40 weekly paintings only insulin treatment and diabetes maintain this high level of tumours, while even medication with methylthiouracil or L-thyroxine fail to do so.

The rather rare granular myoblastomas are found in intact animals only, particularly after administration of insulin or growth hormone and in diabetics though not in rats refractory to alloxan-induced diabetes. Similarly sarcomas and fibromas of the vulva are rare as compared with the dorsal skin (Glucksmann and Cherry, 1971), but occur more often in intact than spayed animals. Basal-celled vulval tumours tend to be slightly more frequent in intacts than in castrates. They are increased very significantly in failed diabetics particularly in intacts and suppressed in castrates given methylthiouracil (Glucksmann and Cherry, 1970*b*). The percentage of induced squamous-celled tumours of the vulva is not greatly influenced by castration or additional medications, but is reduced if the weekly applications are limited to 5 or 10.

Weekly applications of DMBA for life elicit a high percentage of cervico-vaginal sarcomas in intacts, a low one in castrates and an even lower one of epithelial tumours at this site in all animals. It is thus not difficult to reduce formation of connective tissue tumours in intacts and promote them in castrates rather than produce the opposite effects. For the same reason epithelial tumours can be increased rather than reduced. Five groups of additional treatments have been tried to find an adequate explanation for the effect of castration on the induction of sarcomas and the failure of oestrogens to compensate for it:

(1) Procedures affecting the target tissues: oestrogens in various regimes, progesterone, pregnancy, testosterone, adrenalectomy in castrates (to remove residual steroid production), cholesterol as control for oestradiol pellets in cholesterol;

(2) Reduction of immunological competence: cortisone, pelvic and whole body X-irradiation;

(3) General and specific metabolic changes: L-thyroxine, methylthiouracil, growth hormone, insulin and diabetes;

(4) Effect on regulatory centres by perinatal treatments with testosterone, oestrogens, cortisone, L-thyroxine and methylthiouracil;

(5) Variation in carcinogenic dosage by reducing the number of weekly doses of DMBA to 5, 10 or 20 and in castrates additional medication with L-thyroxine or methylthiouracil.

The effects of the various treatments on carcinogenesis in the cervico-vaginal tract of intacts and castrates are summarized in Table IV in comparison with those of weekly doses of DMBA given for life and no additional treatment of the animals. The total number of different additional procedures is 26 for intacts and 29 for castrates. It is quite evident that castrates and intacts are affected differently by the same treatments and that in either group of animals the stimulating effects of additional treatments on sarcoma formation may not be equalled by those on epithelial tumours.

TABLE IV.—*Changes in the Incidence of Cervico-vaginal Tumours*

| Sarcomas | Epitheliomas | Treatments |
|---|---|---|
| Up | Up | ♀ none<br>♀̸ X-rays: pelvic and whole body; cortisone; cholesterol; intermittent stilboestrol; testosterone; methylthiouracil ± stilboestrol; growth hormone; insulin; diabetes |
| Up | Equal | ♀ none<br>♀̸ adrenalectomy; progesterone + oestrogen; testosterone + stilboestrol; L-thyroxine ± stilboestrol; non-diabetics |
| Down | Equal | ♀ oestrogens; 5, 10 or 20 × DMBA; X-rays: pelvic and whole body; methylthiouracil + L-thyroxine; perinatal treatments with: testosterone, L-thyroxine, methylthiouracil, cortisone<br>♀̸ none |
| Down | Up | ♀ progesterone; growth hormone; perinatal treatment with oestrogen<br>♀̸ 5 and 10 × DMBA ± L-thyroxine or methylthiouracil |
| Equal | Up | ♀ methylthiouracil ± stilboestrol<br>♀̸ methylthiouracil + L-thyroxine; 20 × DMBA ± L-thyroxine or methylthiouracil |
| Equal | Equal | ♀ testosterone ± stilboestrol; pregnancy; intermittent stilboestrol; L-thyroxine ± stilboestrol; cortisone; insulin; diabetes; non-diabetics<br>♀̸ oestrogens; progesterone |

In intacts growth hormone reduces the incidence of sarcomas but increases that of epithelial tumours, while in castrates the rate of formation of both types of tumours is enhanced and accelerated. Pelvic or whole body X-irradiation sterilizes intacts and inhibits carcinogenesis while it promotes it in castrates. In spayed animals the induction of sarcomas is promoted by 17 of the 29 procedures listed in Table IV and reduced only by lower dosage of DMBA, while none of the 26 additional treatments applied to intacts promotes and 11 inhibit the formation of sarcomas. The rate of sarcogenesis in intacts is not maximal as shown by comparison with castrates medicated with insulin or alloxan (Fig. 7). Similarly epithelial tumours occur more frequently in castrates with 21 of the 29 additional treatments and only with 4 in intacts. Squamous-celled neoplasms of the vulva are affected only by carcinogenic dosage and not by any of the other additional procedures, while basal-celled tumours at the same site are influenced by agents affecting the growth of the hair follicles.

It remains rather puzzling that castration due to surgery or irradiation causes both atrophy of the cervico-vaginal tract and an inhibition of carcinogenesis, that compensatory growth of the normal structures of the same site elicited by effective doses of oestrogens is not accompanied by an increase in tumour induction, while a variety of other treatments without compensating for the atrophy of the cervico-vaginal tract and irrespective of their effect on gain in body weight promote carcinogenesis in castrates rather than in intacts to a rate exceeding that of intact animals without or with additional treatments. The metabolic changes induced by medication with growth hormone, insulin or alloxan like those with thyroactive compounds affect differently the formation of the same tumour type at different sites, development of different tumour types at the same location and gain in body weight in intact and castrate rats.

This work was supported by grants from the Cancer Campaign for Research.

### REFERENCES

Cherry, C. P. and Glucksmann, A.—(1970) *Br. J. Cancer*, **24**, 510.
Dunn, T. B. and Green, A. W.—(1963) *J. natn. Cancer Inst.*, **31**, 425.
Garvie, W. H. H.—(1969) *Br. J. Cancer*, **22**, 128.
Glucksmann, A. and Cherry, C. P.—(1958) *Br. J. Cancer*, **12**, 32.—(1968) *Br. J. Cancer*, **22**, 545.—(1970*a*) *Br. J. Cancer*, **24**, 333.—(1970*b*) *Br. J. Cancer*, **24**, 769.—(1971) *Br. J. Cancer*, **25**, 735.
Goranson, E. S., Botham, F. and Willms, M.—(1954) *Cancer Res.*, **14**, 730.
Goranson, E. S. and Tilser, G. J.—(1955) *Cancer Res.*, **15**, 626.
Jehl, J., Mayer, J. and McKee, R. W.—(1955) *Cancer Res.*, **15**, 341.
Moon, H. D., Simpson, M. E., Li, C. H. and Evans, H. M.—(1950*a*) *Cancer Res.*, **10**, 297.—(1950*b*) *Cancer Res.*, **10**, 364.
Reuber, M. D.—(1968) *Cancer Res.*, **28**, 2177.
Rosen, F., Budnick, L. E., Solomon, D. K. and Nichol, C. A.—(1961) *Cancer Res.*, **21**, 620.
Salzberg, D. A. and Griffin, A. C.—(1952) *Cancer Res.*, **12**, 294.
Takakura, K., Yamada, H. and Hollander, V. P.—(1967) *Cancer Res.*, **27**, 2034.